# Preoperative Chemoradiotherapy *Versus* Postoperative Chemoradiotherapy for Patients With Locally Advanced Gastric Cancer: A Retrospective Study Based on Propensity Score Analyses

**DOI:** 10.3389/fonc.2020.560115

**Published:** 2020-10-16

**Authors:** Wang Yang, Menglong Zhou, Guichao Li, Lijun Shen, Yan Wang, Hui Zhang, Yaqi Wang, Jing Zhang, Xiaoyang Sun, Zhiyuan Zhang, Wei Zou, Zhen Zhang

**Affiliations:** Department of Radiation Oncology, Fudan University Shanghai Cancer Center, Shanghai, China

**Keywords:** preoperative, postoperative, chemoradiotherapy, gastric cancer, propensity score, survival analysis

## Abstract

**Purpose:**

Although adjuvant chemoradiotherapy (CRT) used to be recommended as a standard of care for locally advanced gastric cancer, this suggestion has been strongly challenged recently. However, clear evidence regarding the optimization of radiotherapy is lacking. The purpose of this study was to compare the effectiveness of preoperative CRT *versus* that of postoperative CRT for resectable or potentially resectable gastric cancer.

**Methods:**

From January 2005 to December 2017, patients with clinical stage III/IVa (*i.e.*, T3-4aN+M0 or T4bNxM0) locally advanced gastric cancer were retrospectively identified. Survival after preoperative CRT and postoperative CRT was assessed by unadjusted, propensity score matching (PSM) and inverse probability of treatment weight (IPTW) analyses. Moreover, exploratory subgroup analyses were performed, and toxicity and patterns of failure were also investigated.

**Results:**

The median follow-up time was 32.5 months. A total of 82 and 463 patients were enrolled in the preoperative and postoperative CRT groups, respectively. After propensity score matching, preoperative CRT was associated with improved overall survival (OS) and disease-free survival (DFS) compared with postoperative CRT (3-year OS: 72.6 *vs.* 54.4%, log-rank p = 0.0021; 3-year DFS: 61.7 *vs.* 44.7%, log-rank p = 0.002). The unadjusted and IPTW analyses yielded consistent results. A complete pathologic response was achieved in 13.4% of the preoperative CRT group. Although the incidence of grade 3 or 4 adverse effects and surgical complications were similar between the two groups, significantly fewer patients experienced treatment interruptions or dose reductions due to toxic effects in the preoperative CRT setting than in the postoperative CRT setting (3.7 *vs.* 10.6%, p = 0.049).

**Conclusions:**

Compared with postoperative CRT, preoperative CRT was associated with improved OS and DFS, superior treatment compliance and comparable surgical complications for patients with locally advanced gastric cancer. Our findings provide important evidence for the optimal combination modalities of surgery and CRT in the absence of randomized clinical data.

## Introduction

Despite a declining incidence, gastric cancer remains the fifth most common malignant cancer and the third leading cause of cancer-related death worldwide (1). Locoregional recurrence and distant metastasis rates have been reported to be high as 21.8–63.4%, even after radical resection (2–4). Accordingly, investigators have explored a variety of multimodality strategies for locally advanced gastric cancer to reduce the relapse rate, including radical surgery plus adjuvant chemoradiotherapy (CRT), preoperative CRT, and perioperative chemotherapy (ChT). The benefit of adjuvant CRT was initially demonstrated by the Intergroup Trial 0116, which reported a distinct survival benefit from concurrent chemoradiation (3, 5). However, a phase III randomized trial known as ARTIST failed to demonstrate long-term survival benefits in patients after D2/R0 gastrectomy (6), and the interim results of the subsequent ARTIST-II trial reported no additional benefits from CRT in patients with pathologically lymph node-positive gastric cancer (7). Similarly, the phase III randomized CRITICS trial, which compared perioperative ChT with preoperative ChT followed by postoperative CRT, conferred no survival benefit from adding radiation to postoperative ChT after adequate preoperative ChT and surgery (8).

Preoperative ChT for gastric cancer showed a survival benefit in two landmark studies: the MAGIC trial using three courses of chemotherapy prior to surgery and three to follow showing significant improvement in 5-year overall survival (36 *vs* 23%) (9) and the French FFCD 9703 phase III trial, in which a perioperative regimen of cisplatin plus flourouracil was utilized, resulting in a significant improvement of curative resection rate and survival (10). The recent FLOT4 study was considered as another landmark which induced pathological complete response (pCR) of up to 16% (11). In view of the excellent pathological regression rate, the NCCN guideline adopted FLOT as the preferred regimen with a category I recommendation (12).

Several studies were also conducted to illustrate the role of preoperative CRT in patients with resectable gastric carcinoma (13, 14). The long-term results of the POET trial published in 2017 showed a significant reduction in locoregional relapse with the addition of radiotherapy to preoperative treatment in patients with locally advanced adenocarcinomas of the esophagogastric junction (15). Although overall survival showed a trend in favor of preoperative CRT, no statistically significant benefit was observed at the 5-year follow-up (p = 0.055), which may partially be attributed to the early termination of the trial and inadequate sample size (15). Inconsistently, the results from a systematic review and meta-analysis revealed a significant overall survival advantage from the combination of preoperative CRT and surgery compared with preoperative chemotherapy (16).

Numerous ongoing phase III trial comparing preoperative ChT and preoperative CRT (NCT01924819, NCT01815853 and NCT03013010) will provide more evidence regarding the best strategy of neoadjuvant ChT or CRT. However, little powered data regarding the direct comparison between preoperative and postoperative CRT has been presented for locally advanced gastric carcinoma, and thus, the superiority of one treatment strategy over another cannot be ascertained. Given the lack of evidence, we sought to compare preoperative concurrent CRT with the same treatment given postoperatively in patients with resectable or potentially resectable gastric cancer based on propensity score analyses.

## Materials and Methods

### Patient Identification

All consecutive patients between June 1, 2005 and December 31, 2017 who met the following eligibility criteria were retrospectively identified: 1) histologically confirmed gastric or gastroesophageal junction cancer; 2) clinical stage III/IVa disease (*i.e.*, T3-4aN+M0 or T4bNxM0) according to the 8^th^ edition of the American Joint Committee on Cancer TNM staging (17); 3) underwent radical R0 gastrectomy and D1/D2 lymphadenectomy; and 4) received preoperative or postoperative CRT. The exclusion criteria included the following: 1) patients with gross or microscopic residual neoplasms at the resection margin; 2) patients who underwent surgery with palliative intent; 3) patients who were lost to follow-up immediately after discharge from the hospital; and 4) patients who had double primary malignancies.

### Treatment Delivery

All patients underwent a total or subtotal gastrectomy and D1/D2 lymphadenectomy with negative margins confirmed by pathology (R0 resection). Radiotherapy was delivered with 6 MV photons using either three-dimensional conformal radiotherapy (3D-CRT) or intensity-modulated radiation therapy (IMRT) preoperatively or postoperatively. The radiotherapy regimen consisted of a 45–50.4 Gy dose delivered in 25–28 fractions (1.8 Gy/fraction, 5 fractions/week). The concurrent chemotherapy regimens were administered as follows: (1) 225 mg/m^2^/d 5-fluorouracil weekly intravenously; (2) 825 mg/m^2^/d capecitabine bid d1-5 weekly; or (3) 40 mg/m^2^/d S1 bid d1-5 weekly. Within 4–8 weeks of completing two to three courses of induction chemotherapy and concurrent CRT, the preoperative CRT group underwent radical resection and 3–4 courses of adjuvant chemotherapy. Most eligible patients in this group received fluorouracil derivatives and platinum-based drugs, or docetaxel-based triplet regimens in the induction phase of preoperative treatment. For the postoperative CRT group, the ChT regimens in the induction phase before surgery mostly consisted of doublet regimens involving fluorouracil and platinum-based, or triplet regimens involving epirubicin, fluorouracil, and platinum-based drugs. 1–2 courses of adjuvant chemotherapy were administered after surgery, followed by concurrent CRT and 4–5 additional subsequent courses of chemotherapy. Radiotherapy was targeted on the primary tumor site, perigastric tumor extension, involved lymph nodes, and elective lymph node stations for the preoperative CRT group (18) and tumor bed, anastomosis site, duodenal stump, and elective regional lymph nodes for the postoperative CRT group (19).

Adverse events were assessed using the Common Terminology Criteria for Adverse Effect (CTCAE) version 5.0 (20).

### Follow-up and Definition of Recurrence

After completing primary treatment, the patients were followed up in three-month intervals for the first two years, six-month intervals until five years, and annually thereafter. The standardized evaluation consisted of a physical examination, laboratory tests, computed tomography (CT), or magnetic resonance scans of the chest, abdomen and pelvis at each visit and endoscopy each year.

Local recurrence was defined as any relapse at the site of anastomosis, duodenal stump, tumor bed, or remnant stomach. Regional recurrence was defined as recurrence involving the regional lymph nodes. Peritoneal dissemination was considered to include metastasis inside the peritoneal cavity, such as the peritoneum, colorectum, ovary, and ureter. Distant metastasis was defined as metastasis to distant organs or extra-abdominal lymph nodes.

### Statistical Analysis

The primary objective of this study was to compare overall survival (OS) and disease-free survival (DFS) after preoperative CRT versus postoperative CRT. OS was considered from the time of diagnosis to date of death from any cause or last follow-up contact. DFS was calculated from the time of diagnosis to initial failure or the day of last follow-up without recurrence. The second objective was to compare the failure patterns, toxicities, and compliance rates.

Data are presented as the mean (± SD) for continuous variables and numbers (percentages) for categorical variables. Patient demographic and clinical characteristics were compared using the chi-square test or independent sample t-test/Wilcoxon rank sum test, as appropriate.

Given the difference in the baseline characteristics between the two groups (**Table 1**), propensity score analyses, including propensity score matching (PSM) and inverse probability of treatment weight (IPTW) analyses, were applied. The propensity scores were developed with a logistic regression model to account for potential confounding factors. Clinically relevant variables, including sex, age, tumor location, T stage and N stage, which may be associated with survival or treatment assignment, were included in the propensity score model. Patients treated with preoperative CRT were matched 1:2 to patients treated with postoperative CRT based on propensity score using an optimal matching algorithm. The IPTW was then calculated with the estimated propensity scores. In the pseudo data using IPTWs, the number of observations is the sum of the weights (21). The OS and DFS of the original patients, matched patients and IPTW estimators were plotted by using the Kaplan–Meier method and compared with log-rank tests. Within the matched group, interaction and subgroup analyses were conducted to assess the heterogeneity of treatment effects. All statistical analyses were performed using the R statistical software package, version 3.6.0 (R Project for Statistical Computing, Vienna, Austria). A two-sided significance level of 0.05 was applied.

**Table 1:** Baseline demographic and clinical characteristics of patients with gastric cancer in the original cohort.

Characteristics	Preoperative CRT (n = 82)	Postoperative CRT (n = 463)	P value
Sex			0.751
Male	55 (66.3%)	322 (69.5%)	
Female	27 (32.9%)	141 (30.5%)	
Age (years)			
Mean (SD)	57.3 (10.9)	52.9 (10.7)	**0.001**
Primary location			0.666
Upper 1/3	26 (31.7%)	125 (27.0%)	
Middle 1/3	24 (29.3%)	150 (32.4%)	
Lower 1/3	32 (39.0%)	188 (40.6%)	
Initial T stage			**<0.001**
T3	5 (6.1%)	207 (44.7%)	
T4a	60 (73.2%)	229 (49.5%)	
T4b	17 (20.7%)	27 (5.8%)	
Initial N stage			**0.010**
N0	1 (1.2%)	2 (0.4%)	
N1	19 (23.2%)	75 (16.2%)	
N2	34 (41.5%)	149 (32.2%)	
N3a	26 (31.7%)	168 (36.3%)	
N3b	2 (2.4%)	69 (14.9%)	
Lymphadenectomy			
D0/D1	8 (9.8%)	61 (13.2%)	0.498
D2	74 (90.2%)	402 (86.8%)	
Follow-up period (months)	45.0 (35.4)	33.7 (22.2)	**0.005**

CRT, chemoradiotherapy.

Values are presented as number (%) or mean ± standard deviation.

The P < 0.05 was considered statistically significant and was represented as bold.

## Results

### Study Population

**Figure 1:**
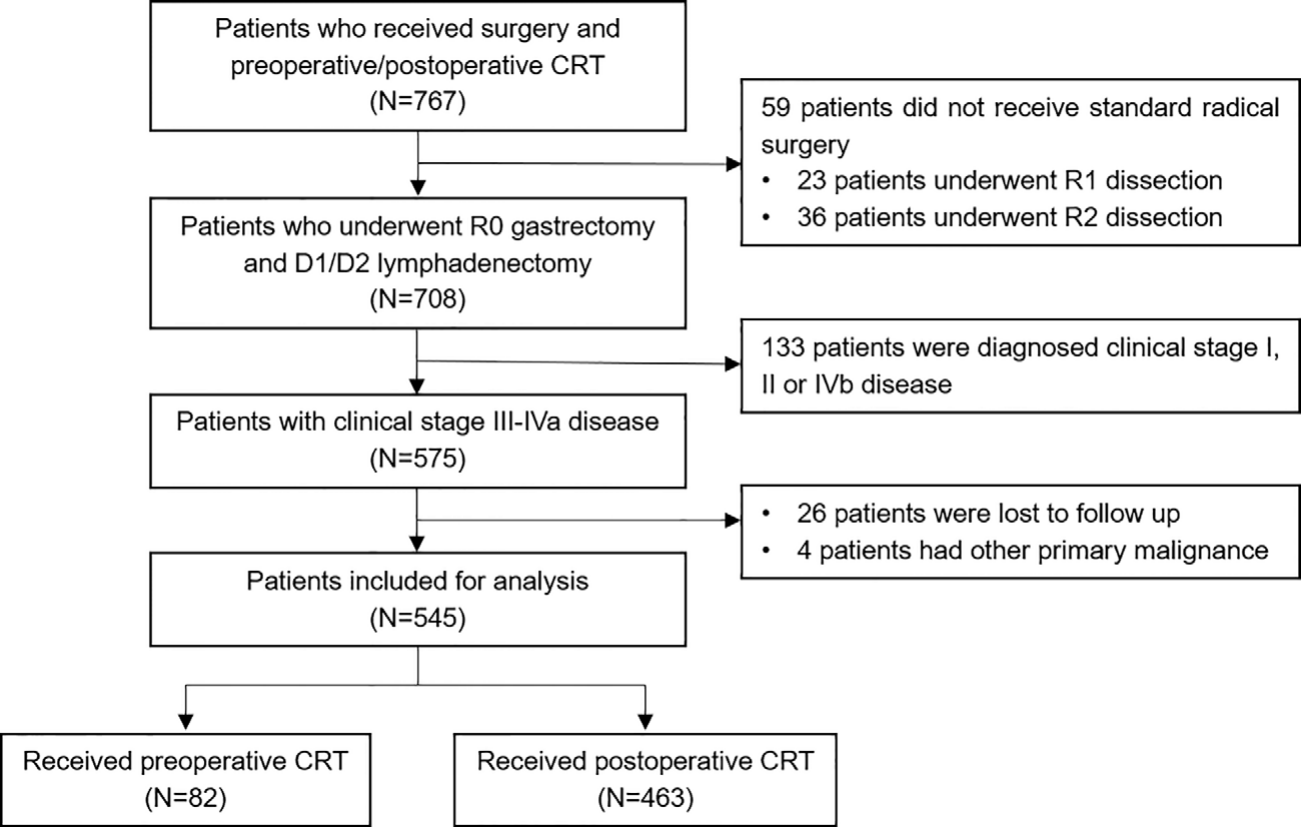
Flow diagram of the patient selection process based on the eligibility criteria and exclusion criteria. CRT, chemoradiotherapy.

**Figure 2:**
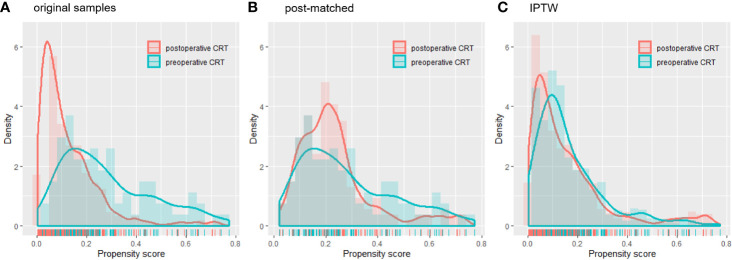
Histograms and density curves depicting the **(A)** original patients, **(B)** post-matched cohort and **(C)** IPTW propensity score distribution in the preoperative CRT and postoperative CRT groups. CRT, chemoradiotherapy; IPTW, inverse probability of treatment weight.

A total of 767 patients who had undergone surgery and preoperative/postoperative CRT were identified. Of these, 222 individuals were excluded, and a detailed flow diagram of the patient inclusion process is shown in **Figure 1**. Ultimately, 82 and 463 patients who received preoperative CRT and postoperative CRT as a treatment modality met the criteria and were included in the analysis. The baseline clinicopathological characteristics of the original patients are summarized in **Table 1**. Compared with the patients in the postoperative CRT group, patients who received preoperative CRT were older (p = 0.001) and more likely to have more advanced disease (T stage, P < 0.001; N stage, p = 0.010). Since preoperative CRT was mainly applied after 2012, the follow-up period of the postoperative CRT cohort was longer than that of the preoperative CRT cohort (p = 0.005). Matching and IPTW analyses with propensity scores achieved balance between two groups for all variables (**Table 2**). Histograms and density graphs depicting comparisons of the original, post-matched and post-weighted propensity score distributions of each group are shown in **Figure 2**.

**Table 2:** Baseline demographic and clinical characteristics of patients in the propensity score-matched cohort and inverse probability of treatment-weighted cohort.

Characteristics	Propensity 1:2 Matching (n = 246)			IPTW (n = 540)		
	Preoperative CRT (n = 82)	Postoperative CRT (n = 164)	P value	Preoperative CRT (n = 73)	Postoperative CRT (n = 467)	P value
Sex			0.521			0.366
Male	55 (66.3%)	118 (72.0%)		54 (73.5%)	321 (68.7%)	
Female	27 (32.9%)	46 (28.0%)		19 (26.5%)	146 (31.3%)	
Age (years)						
Mean (SD)	57.3 (10.9)	57.2 (8.0)	0.964	52.9 (11.8)	53.5 (10.5)	0.654
Primary location			0.994			0.334
Upper 1/3	26 (31.7%)	52 (31.7%)		26 (36.0%)	130 (27.8%)	
Middle 1/3	24 (29.3%)	49 (29.9%)		19 (25.5%)	152 (32.5%)	
Lower 1/3	32 (39.0%)	63 (38.4%)		28 (38.5%)	185 (39.7%)	
T stage at diagnosis			0.266			0.070
T3	5 (6.1%)	19 (11.6%)		15 (20.3%)	144 (30.8%)	
T4a	60 (73.2%)	120 (73.2%)		54 (73.3%)	275 (58.9%)	
T4b	17 (20.7%)	25 (15.2%)		5 (6.3%)	48 (10.2%)	
N stage at diagnosis			0.295			0.118
N0	1 (1.2%)	2 (1.2%)		1 (1.0%)	3 (0.7%)	
N1	19 (23.2%)	42 (25.6%)		9 (12.7%)	88 (18.9%)	
N2	34 (41.5%)	60 (36.6%)		27 (37.3%)	149 (32.0%)	
N3a	26 (31.7%)	44 (26.8%)		32 (44.0%)	161 (34.6%)	
N3b	2 (2.4%)	16 (9.8%)		4 (5.1%)	64 (13.8%)	
Lymphadenectomy			0.625			0.121
D0/D1	8 (9.8%)	21 (12.8%)		5 (7.1%)	62 (13.3%)	
D2	74 (90.2%)	143 (87.2%)		68 (92.9%)	405 (86.7%)	

IPTW, inverse probability of treatment weight; CRT, chemoradiotherapy.

### Pathological Evaluation and Surgical Procedures

The details of the postoperative pathological stage and type of surgery in the post-matched cohorts are listed in **Table 3**. After preoperative CRT, a shift towards earlier T stage occurred in 82.9% of the patients and N stage in 72.0% of the patients. A total of 13.4% of the patients had a pCR, and only 48.8% of the patients had positive lymph nodes in the preoperative CRT group. The rate of D2 lymphadenectomy did not differ between the two groups when 246 matched patients were considered (p = 0.625).

**Table 3:** Postoperative pathological tumor stage and type of surgery in matched samples.

Variable	Preoperative CRT (n = 82)	Postoperative CRT (n = 164)	P value
Pathological stage			**<0.001**
pCR	11 (13.4%)	0 (0.0%)	
I	21 (25.6%)	0 (0.0%)	
II	28 (34.1%)	12 (7.3%)	
III	22 (26.8%)	152 (92.7%)	
Differentiation			
Poor	36 (43.9%)	135 (82.3%)	**<0.001**
Well-moderate	14 (17.1%)	16 (9.8%)	
Not specified	32 (39.0%)	13 (7.9%)	
Vessel			
Positive	22 (26.8%)	89 (54.3%)	**<0.001**
Negative	60 (73.2%)	55 (33.5%)	
Not specified	0 (0.0%)	20 (12.2%)	
Nerve			
Positive	21 (25.6%)	112 (68.3%)	**<0.001**
Negative	61 (74.4%)	32 (19.5%)	
Not specified	0 (0.0%)	20 (12.2%)	
No. of involved lymph nodes			**<0.001**
Mean (SD)	2.0 (3.0)	6.9 (6.4)	
Median (Range)	0.5 (0-13)	5 (0-31)	
No. of lymph nodes dissected			**0.023**
Mean (SD)	19.4 (7.5)	22.3 (10.5)	
Median (Range)	18 (7-55)	21 (5-65)	
Lymphadenectomy			
D0/D1	8 (9.8%)	21 (12.8%)	0.625
D2	74 (90.2%)	143 (87.2%)	

CRT, chemoradiotherapy; pCR, pathological complete response.

The P < 0.05 was considered statistically significant and was represented as bold.

### Treatment Delivery and Safety

Treatment compliance and toxicity data were collected for all unmatched patients. Patients were treated with a median radiation dose of 45 Gy in both groups. In the original sample, significantly fewer patients experienced interruptions or dose reductions for concurrent chemotherapy due to toxic effects in the preoperative CRT group than in the postoperative group (3.7 *vs.* 10.6%, p = 0.049), whereas an interruption in radiation was rare in both groups (1.2 *vs.* 3.2%, p = 0.52). In the pre- and postoperative CRT groups, 1.2 and 21.3% of patients, respectively, received radiation without concurrent chemotherapy during the course of radiotherapy after considering the toxicities and tolerability of therapy.

The adverse events induced by chemoradiation are summarized in **Table 4**. In general, preoperative and postoperative CRT were both well tolerated. The most common non-hematologic toxicities included anorexia, nausea, vomiting and hepatic dysfunction. The overall rates of grade 3 or 4 side effects were similar between the two groups, and leucopenia/neutropenia was more likely to occur in patients treated with the postoperative approach than those treated with the preoperative approach. No treatment-related deaths occurred. The postoperative complications were ileus (n = 2), anastomotic leakage (n = 1), bleeding (n = 1) and intra-abdominal infection (n = 1) in the preoperative CRT group and infection (n = 2), anastomotic leakage (n = 1) and gastroplegia (n = 1) in the postoperative CRT group.

**Table 4:** Adverse events of chemoradiotherapy.

Toxicities	Preoperative CRT (n = 82)		Postoperative CRT (n = 463)	
	Any grades	Grade 3 or 4	Any grades	Grade 3 or 4
Hematotoxicity				
Leucopenia	58 (70.7%)	7 (8.5%)	397 (85.7%)	38 (8.2%)
Neutropenia	28 (34.1%)	3 (3.7%)	252 (54.4%)	21 (4.5%)
Thrombocytopenia	40 (48.8%)	4 (4.9%)	216 (46.7%)	19 (4.1%)
Anemia	52 (63.4%)	4 (4.9%)	259 (55.9%)	7 (1.5%)
Gastrointestinal tract				
Anorexia	41 (50.0%)	1 (1.2%)	236 (51.0%)	19 (4.1%)
Nausea	25 (30.5%)	0 (0.0%)	153 (33.0%)	17 (3.7%)
Vomiting	14 (17.1)	0 (0.0%)	68 (14.7%)	9 (1.9%)
Hepatic dysfunction	26 (31.7%)	0 (0.0%)	151 (32.6%)	4 (0.9%)
Renal dysfunction	4 (4.9%)	0 (0.0%)	8 (1.7%)	0 (0.0%)

### Survival and Subgroup Analysis

**Figure 3:**
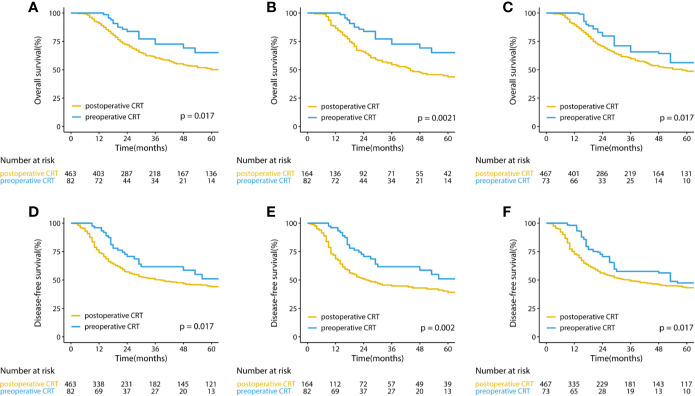
Kaplan–Meier curves for overall survival and disease-free survival in **(A, D)** the original cohort and **(B, E)** the cohorts after PSM and **(C, F)** IPTW-adjusted analyses of patients with gastric cancer. CRT, chemoradiotherapy; PSM, propensity score matching IPTW, inverse probability of treatment weight.

**Figure 4:**
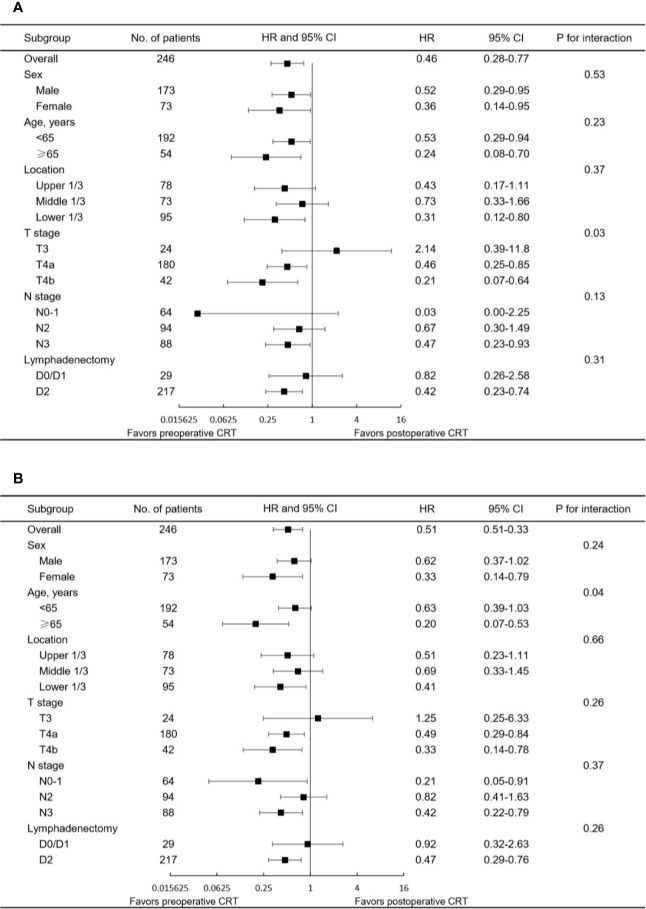
Forest plot of the subgroup analysis for **(A)** OS and **(B)** DFS in the matched study population. HR<1 favors preoperative CRT, and HR>1 favors postoperative CRT. CRT, chemoradiotherapy; CI, confidence interval; HR, hazard ratio.

The median follow-up for the entire cohort was 32.5 months (interquartile range, 18.2 to 64.9 months). In the multivariate analysis of the pre-matched dataset, preoperative CRT was associated with significant benefits for OS (p = 0.002) and DFS (p = 0.001). Survival curves of OS and DFS are shown in **Figure 3**, and the three datasets, including the original patients and patients after PSM and IPTW analysis, revealed similar results. Among the post-matched sample, the overall survival rates at 1, 3, and 5 years were 100, 72.6, and 65.1% in the preoperative CRT group and 86.6, 54.4, and 43.7% in the postoperative CRT group, respectively (log-rank p = 0.0021). The 1-, 3-, and 5-year DFS rates were 96, 61.7, and 51% in the preoperative CRT arm *versus* 68.6, 44.7, and 39.2% in the postoperative CRT arm, respectively (log-rank p = 0.002). In the exploratory subgroup analysis of the post-matched cohort, the benefit of preoperative CRT for OS and DFS was consistent across most subgroups examined. Heterogeneity of the treatment effect remained in the subgroup defined by initial T stage for OS and age for DFS among the tumor-specific covariate levels (**Figures 4****)**.

### Patterns of Failure

During the follow-up period, 19 patients (23.2%) and 69 patients (42.1%) in the pre- and postoperative CRT groups experienced relapse, respectively; of these cases, peritoneal and distant metastasis were most commonly observed, regardless of the pre- or postoperative CRT group (**Table 5**). The overall rates of recurrence were lower with the preoperative approach than with the postoperative approach, especially with respect to local recurrence, peritoneal metastasis and distant metastasis.

**Table 5:** Patterns of total recurrence over the entire follow-up period.

Recurrence Site	Preoperative CRT (n = 82)	Postoperative CRT (n = 164)
Local recurrence	2 (2.4%)	7 (8.5%)
Regional failure	9 (11.0%)	19 (11.6%)
Peritoneal metastasis	11 (13.4%)	30 (18.3%)
Distant metastasis	10 (12.2%)	30 (18.3%)

## Discussion

The rationale for preoperative CRT is to treat locoregional micrometastases as early as possible, downstage advanced tumors, enhance the rate of curative resection, and improve treatment compliance. Furthermore, irradiation may be more effective preoperatively since tumor oxygenation is better with the preoperative approach than in the postoperative setting. Multiple trials evaluating preoperative CRT have shown its advantages in terms of pCR, local control, R0 resection rate or survival rate (15, 16, 22). In the German POET trial, patients with locally advanced adenocarcinomas of the esophagogastric junction were randomized to ChT or induction ChT and CRT followed by surgery. Benefits in terms of pCR (14.3 *vs.* 1.9%, p = 0.03) and local progression-free survival (p = 0.01) were reported in the CRT group relative to the ChT group (13). Moreover, updated long-term data published in 2017 showed a trend in favor of preoperative CRT for overall survival (p = 0.055) (15). The ongoing international phase III TOPGEAR trial included patients with resectable adenocarcinoma of the stomach or gastroesophageal junction and compared perioperative ChT and induction ChT followed by CRT. In the interim analysis of this study, preoperative CRT showed good treatment compliance and acceptable tolerance, indicating that this modality could be delivered safely and feasibly (14).

Although there have been several studies demonstrating the advantages of preoperative

CRT for esophageal/gastroesophageal junction cancer, few comparative data of pre- and postoperative chemoradiotherapy in locally advanced gastric cancer have been reported to date. The current study identified the superiority of delivering radiotherapy preoperatively rather than postoperatively in patients with resectable or potentially resectable gastric cancer based on propensity score analyses. Significant advantages in OS and DFS were achieved in patients treated with preoperative CRT, and these benefits remained consistent and stable after PSM and IPTW analyses were performed. In the post-matched cohort, 3-year OS and DFS rates of 72.6 and 61.7%, respectively, were reported in the preoperative CRT arm, showing superior outcomes than those reported in previous trials (15). However, these data should be interpreted cautiously, since all patients enrolled in this study underwent R0 resection because of its retrospective design. The results of the explorative subgroup analysis indicated that most subgroups favored preoperative CRT but more advanced tumors (*i.e.*, stage T4 disease) may benefit more than less advanced tumors.

In contrast to the INT-0116 adjuvant CRT trial, in which 17% of the patients stopped treatment due to treatment-related toxicity (23), 89.4% of the patients in the postoperative CRT group were able to complete concurrent CRT as planned in the current study. Pleasingly, giving radiation preoperatively further improved treatment compliance, and only 3.7% of the patients experienced interruptions or dose reductions because of toxic effects, which is similar to the rate reported in the TOPGEAR trial (14). In addition, the overall toxicity of preoperative and postoperative modality therapy was similar, and the incidence of leucopenia/neutropenia was even slightly lower in patients treated with the preoperative approach than in those treated with the postoperative approach. A major concern among surgeons with respect to preoperative therapy may be the negative effect of surgical complications. Fortunately, the results in our study contradicted that postponing surgery for preoperative CRT plus ChT would allow tumor regression and did not result in an increased rate of surgical complications.

With the increasing administration of preoperative treatment, an accurate preoperative evaluation of tumor invasion depth and lymph node metastasis is crucial to making an optimal therapeutic decision. In diagnosing tumor invasion, the accuracy of CT was 65 to 92% and that of EUS was 77.1 9to 88.8%. For N staging, the sensitivity of nodal invasion is reported to range from 62 to 92% using axial or multidetector CT (24). A review including 44 studies reported a pooled sensitivity of 83% and specificity of 95% for patients with gastric cancer (25). To summarize, no staging method delivers both high sensitivity and specificity so far, resulting in difficult preoperative staging. In the current study, initial clinical staging was performed preoperatively by CT, EUS and diagnostic laparoscopy. A total of 91.4% of the patients in the preoperative CRT group underwent laparoscopic exploration to avoid unnecessary surgery and radiation, since previous publications have demonstrated the superiority of laparoscopy over other modalities for the detection of peritoneal metastases (26).

This is the first study, to the best of our knowledge, to compare pre- and postoperative CRT directly in locally advanced gastric cancer. Despite the retrospective design of the study, we conducted propensity score analyses to mitigate selection bias and immortal time bias. However, there remain several limitations to the current study. First, some patients receiving preoperative treatment might have been misstaged due to the lack of accurate preoperative staging methods. Second, information with respect to Lauren’s classification was not included in this study due to missing data, although it was considered a crucial prognostic factor in published studies. Third, the cases in the postoperative CRT group were retrieved over a long time period; in contrast, preoperative CRT has just become an optional strategy in recent years. Thus, changes in systemic chemotherapy regimens may have an impact on outcomes. In addition, this study was limited by its retrospective design, and all patients identified in this study underwent R0 gastrectomy, which may lead to an overestimation of long-term survival in both groups.

## Conclusions

In conclusion, compared with postoperative CRT, preoperative CRT was associated with improved OS and DFS, superior treatment compliance and comparable surgical complications.

Therefore, the authors recommended that strategies designed to deliver radiotherapy and chemotherapy prior to surgery are generally well tolerated and may be the preferred treatment for patients with locally advanced gastric cancer. For further validation, data from phase III randomized controlled trials, such as the TOPGEAR trial, are eagerly awaited.

## Data Availability Statement

The raw data supporting the conclusions of this article will be made available by the authors, without undue reservation.

## Ethics Statement

The studies involving human participants were reviewed and approved by the Fudan University Shanghai Cancer Center. The patients/participants provided their written informed consent to participate in this study. Written informed consent was obtained from the individual(s) for the publication of any potentially identifiable images or data included in this article.

## Author Contributions

WY, MZ, GL, and ZZ designed the research. WY, XS, ZYZ, and WZ acquired the data. WY and GL conducted the statistical analysis. WY and MZ wrote the paper. GL, LS, YQW, HZ, and ZZ revised the manuscript. WY, MZ, GL, and ZZ had primary responsibility for final content. All authors contributed to the article and approved the submitted version.

## Funding

This work was supported by the National Natural Science Foundation of China (Grant No 81773357).

## Conflict of Interest

The authors report no conﬂicts of interest in this work.

## Abbreviations

CRT, chemoradiotherapy; PSM, propensity score matching; inverse probability of treatment weight, IPTW; OS, overall survival; DFS, disease-free survival; ChT, perioperative chemotherapy; pCR, pathological complete response; 3D-CRT, 3-dimensional conformal radiotherapy; IMRT, intensity-modulated radiation therapy; CTCAE, Common Terminology Criteria for Adverse Effect; CT, computed tomography.
